# Experiment-based calibration in psychology: Optimal design considerations

**DOI:** 10.1016/j.jmp.2023.102818

**Published:** 2023-12

**Authors:** Dominik R. Bach

**Affiliations:** Wellcome Centre for Human Neuroimaging & Max–Planck UCL Centre for Computational Psychiatry and Ageing Research, University College London, United Kingdom; University of Bonn, Transdisciplinary Research Area ”Life and Health”, Hertz Chair for Artificial Intelligence and Neuroscience, Germany

**Keywords:** Calibration, Retrodictive validity, Measurement uncertainty, Measurement accuracy

## Abstract

Psychological theories are often formulated at the level of latent, not directly observable, variables. Empirical measurement of latent variables ought to be valid. Classical psychometric validity indices can be difficult to apply in experimental contexts. A complementary validity index, termed retrodictive validity, is the correlation of theory-derived predicted scores with actually measured scores, in specifically designed calibration experiments. In the current note, I analyse how calibration experiments can be designed to maximise the information garnered and specifically, how to minimise the sample variance of retrodictive validity estimators. First, I harness asymptotic limits to analytically derive different distribution features that impact on estimator variance. Then, I numerically simulate various distributions with combinations of feature values. This allows deriving recommendations for the distribution of predicted values, and for resource investment, in calibration experiments. Finally, I highlight cases in which a misspecified theory is particularly problematic.

## Highlights

• Experiment-based calibration is a method for validation of psychological measurement.
• Calibration benefits from low variance of sample estimators.
• Equiprobable standard values reduce estimator variance.
• Reducing random aberration does not always reduce estimator variance.
• Systematic aberration is particularly problematic when inverse sigmoid.

## 1 Introduction

Many areas of psychology deal with latent variables that are not directly observable, such as subjective value, confidence, or arousal. Empirical research mandates that such variables are assessed via a suitable measurement procedure. This raises a question of measurement validity: does the measurement actually relate to the latent variable of interest, and if yes, then how close is that relation? A validity argument is usually based on several sources (AERA, Joint Committee on the Standards for Educational, Psychological Testing of the American Educational Research Association, & the American Psychological Association and the National Council on Measurement in Education, 2014), among them quantitative indices such as convergent and discriminant validity, or reliability. While some of these are commonly used, others are less widespread in practice. For example, discriminant validity, while theoretically indispensable for interpretation of convergent validity (McDonald, 2013), is more infrequently assessed (Zumbo & Chan, 2014). This might reflect practical and interpretational challenges in the application of psychometric indices (Bach, Rigdon & Sarstedt, 2023). For a practical example, validity assessment requires demonstrating convergent and divergent validity, which necessitates finding at least two measurement methods for the same, and for a different, latent variable, and this can be challenging. For an interpretational example, when convergent validity is low, then it cannot adjucate between a “more”, and a “less” valid method (Bach, Rigdon et al., 2023). Furthermore, psychometrics is historically grounded in the study of interindividual difference, and all psychometric indices require incidental between-person variability to be meaningful (Brandmaier et al., 2018). In contrast, much of experimental psychology is concerned with general or average treatment effects, where the goal of experimental design is often to minimise variability of the treatment effect between persons (Hedge, Powell, & Sumner, 2018). In such cases, psychometric indices can be misleading.

**Fig. 1:**
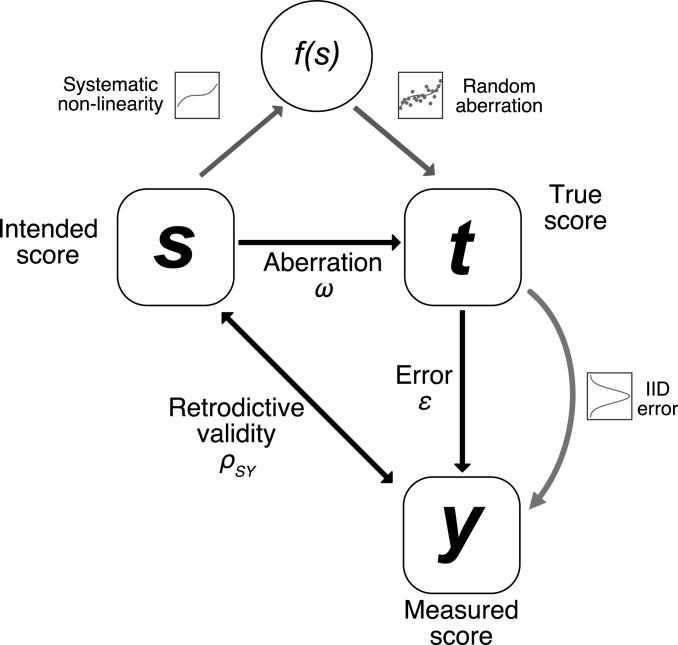
Schematic of the calibration approach. In the analytical derivations, no assumptions are made for the distributions of ω and ɛ (black arrows). In the simulations, aberration ω is decomposed into a non-linearity f and random aberration, and measurement error is assumed to be unbiased i.i.d. for simplicity (grey arrows).

Experiment-based calibration has recently been proposed as a complementary strategy to compare measurement methods, and does not suffer from the aforementioned shortcomings. Inspired by contemporary approaches to the evaluation of physical measurement (Phillips, Estler, Doiron, Eberhardt, & Levenson, 2001), the strategy is to generate well-defined values of a latent variable in a standardised experiment, and evaluate methods by how well they can reproduce these values (Bach, Melinscak, Fleming, & Voelkle, 2020). This is formalised in the concept of retrodictive validity: the correlation of intended scores of the latent variable with the actually measured scores (see Fig. 1) (Bach & Melinscak, 2020). Conceptually, this can be seen as a type of criterion validity, with the external validity criterion replaced by experiment-defined standard scores (Bach et al., 2020).

While previous work has formally laid out the concept of retrodictive validity, and detailed the statistical assumptions under which it is informative on measurement accuracy (Bach et al., 2020), it has not addressed how the concept can be put into practice — or in other words, how a calibration experiment should be designed. There are at least two perspectives on calibration design. The first is domain-specific: to define a suitable experimental manipulation that can be used to impact on a particular latent variable. Recent work has suggested an expert consensus procedure to this end, and has provided an exemplary experiment to calibrate the measurement of associative learning (Bach et al., 2023). The second perspective is statistical: how can numerical and distributional design features be used to improve calibration? This is the topic of the current work.

To clearly define the objective, i.e., what does it mean to “improve calibration”, I draw on statistical estimation theory. Development of experimental measurement methods is often incremental, and previous research in the field of psychophysiology has suggested that the achievable improvements might be relatively small (Bach & Melinscak, 2020). In this situation, the challenge is to distinguish between two relatively similar population values for retrodictive validity from sample estimators. Consequently, retrodictive validity estimates themselves should be accurate. Thus, I ask how design features can be used to maximise accuracy of retrodictive validity estimators. Retrodictive validity is expressed as bivariate correlation. Sample estimators for correlation coefficients are approximately unbiased for large samples, such that the objective of maximising accuracy reduces to minimising estimator variance. Estimator variance, in this case, depends not only on sample size but crucially on features of the parent distributions which will often be non-normal. Hence, the approach taken here is to analytically identify relevant distribution features using asymptotic limits, and then to numerically simulate distributions with combinations of these features in finite samples. Note that all simulation results depict the quantity $nV\left(r_{SY}\right)$ which is (approximately) independent of sample size n. The variance of the retrodictive validity estimator in a finite sample of particular size can be derived from this quantity by dividing through the actual sample size used.

## 2 Methods

### 2.1 Model objects and notation

#### 2.1.1 Overview

To recapitulate the calibration approach, an experiment is conducted in which the independent variable is chosen to generate predicted standard scores S of the latent variable (see Table 1). However, these predicted values are not accurately achieved: the truly achieved scores T deviate by experimental aberration ω. Measurement operations are then performed to quantify T, but the measured scores Y deviate by some error ɛ. Because psychological variables have no scale, we can disregard linear mappings between S, T, and Y without loss of generality. To illustrate this with an example, consider the calibration of lightness measurement (i.e., the perception of an object’s luminance). Experimenters can vary (physical) luminance and colour of an object in order to manipulate (perceived) lightness. Then the standard scores S are defined by a vector of the physical quantities (luminance and colour) together with a formal theory of lightness (e.g. the CIECAM02 model) that takes these quantities as input. A simple measurement operation might consist in asking people to indicate lightness on a visual analogue scale.

Experimental aberration ω has two sources. The first is that the theory that links experimental manipulation with latent variable scores (the CIECAM02 lightness model in our example) might be inaccurate; in other words, S is mis-specified. This is modelled here with a systematic non-linearity $f\left(S\right)$. The second reason is random variation due to imprecision $\omega_{I}$ (due to physical and psychological factors), which is modelled by a (scalable) probability distribution that may depend on S (corresponding to non-additive noise). Measurement error ɛ also has two sources: systematic mis-specification in the measurement system, and random variation.

**Table 1:** Notation used in the methods and results.

E	Expectation

V	Variance

S	Standard (i.e. intended or predicted) scores of the latent variable

T	True (i.e. achieved) scores of the latent variable

Y	Measured scores of the latent variable

ω	Experimental aberration: T=S+ω. Experimental aberration can be decomposed into aberration due to non-linearity, and random (imprecision) aberration: $\omega =\left(S-f\left(S\right)\right)+\omega_{I}$, see next row.

f, $\omega_{I}$	Non-linearity and random (imprecision) aberration: $T=f\left(S\right)+\omega_{I}\left(S\right)$, where f:R→R is fixed for a given value of S, and $\omega_{I}$ is randomly drawn from a probability distribution that depends on S.

ɛ	Measurement error: Y=T+ɛ

$\rho_{SY}$	Retrodictive validity (i.e. correlation between standard and measured attribute scores)

$r_{SY}$	Retrodictive validity estimator (i.e. sample correlation between standard and measured attribute scores)

$\zeta_{I}\in R^{+}$, $\omega_{0}$	Scale and shape of random (imprecision) aberration: $\omega_{I}=\zeta_{I}\omega_{0}$, $V\left(\omega_{0}\right)=1$, $\zeta_{I}^{2}=V\left(\omega_{I}\right)$

$\xi \in R^{+}$	Scaling constant in various simulations

$\sigma^{2}\in R^{+}$	Variance of normal distributions used in various simulations

#### 2.1.2 Standard values

Standard values S are the experimenters’ prediction for the effect of their experimental manipulation on the latent variable. Since experimenters have full control over the experimental manipulation, the distribution of S is a key consideration for designing a calibration experiment. In some cases where the theory is well-developed (e.g. CIECAM02 model for lightness perception), S might be chosen from a large range, allowing many different distributions including approximately normal distributions. In many other cases, the theory that links independent variable to latent attribute values might not be numerically specified and only allow predictions for a discrete number of values of the independent variable. For example, attributes like “fear memory” or “confidence” might only allow predicting two standard values, i.e. “low” and “high” (Bach et al., 2023). In the numerical simulations, we consider discrete and continuous distributions, including a standard normal distribution, and a truncated normal distribution, where the latter is more feasible for experimental implementation.

#### 2.1.3 Experimental aberration

The distribution of the experimental aberration ω is not under direct experimental control. First, if the systematic non-linearity $f\left(S\right)$ were precisely known then one would be able to include it in the model and thus adjust the predicted standard values S. Secondly, precisely revealing the distribution of random aberration would already require a perfect measurement system. However, it is still useful to consider the impact of these two terms. Regarding the non-linearity, theoretical or experimental considerations might suggest a particular class of non-linearity $f\left(S\right)$. For example, the Helmholtz–Kohlrausch effect, which is not included in the CIECAM02 model, would suggest that for certain colours, the model over- or underestimates the true perceived lightness, even if this predicted mis-specification is not known numerically. Regarding random aberration, it may be possible to unspecifically reduce the scale of the aberration with experimental means. For example, conducting a psychophysics study in a dedicated lab instead of as an online experiment is likely to result in smaller aberration. This is likely to include an impact on the shape of aberration distribution, which is unknown. Here, we model this situation with an unspecific impact on the scale of random aberration. This is why we decompose random aberration $\omega_{I}=\zeta_{I}\omega_{0}$ into shape $\omega_{0}$, and scale $\zeta_{I}\in R$ with $V\left(\omega_{0}\right)≔1$ and $V\left(\omega_{I}\right)=\zeta_{I}^{2}$.

#### 2.1.4 Measurement error

Measurement error is not under direct experimental control in a calibration experiment. Crucially, data transformations after the experiment may influence measurement error and thus are not known a priori. Hence, a calibration experiment should be designed in a way that is useful under various levels of measurement error. In numerical simulations, we assume throughout that aberration and measurement error are not only uncorrelated but also stochastically independent, and that measurement error follows a normal distribution. We explore several levels of error variance in order to account for measurement instruments of different precision. To facilitate the results and because it is difficult to foresee a priori, we do not consider the effect of systematic measurement error.

#### 2.1.5 Scaling

Because psychological variables have no natural scale or anchor point, we can assume, without loss of generality: $E\left(S\right)=E\left(T\right)=E\left(Y\right)=E\left(\omega \right)=E\left(ɛ\right)=0$, $V\left(S\right)=1$, $Cov\left(S,T\right)=1$.

### 2.2 Numerical simulations

In our simulations, we evaluate the following distributions. These examples are based on the analytical results and represent distributions with extreme values for some relevant features. For simplicity, the simulations consider calibration experiments in which the standard scores are independently randomised for each instantiation of the experiment, rather than being balanced.

#### 2.2.1 Standard values

1. Two equiprobable values: $S\in \left\{-1,1\right\}$.
2. Four equiprobable values, ensuring $V\left(S\right)=1$: $S\in \left\{-3/\sqrt{5},\right)\left(-1/\sqrt{5},1/\sqrt{5},3/\sqrt{5}\right\}$.
3. Continuous uniform distribution, ensuring $V\left(S\right)=1$: $S∼U\left(-\sqrt{3},\sqrt{3}\right)$.
4. Standard normal distribution: $S\in R,\;S∼N\left(0,1\right)$.
5. Truncated normal distribution with variance $\sigma^{2}$ numerically determined to ensure $V\left(S\right)=1$: $S∼N\left(0,\sigma^{2}\right),S\in \left[-2\sigma ,2\sigma \right]$.

#### 2.2.2 Systematic non-linearity in the experimental aberration

For the simulated non-linearities $f\left(S\right)$ (see Table 1), the parameter ξ is numerically determined to ensure that the linear component of the non-linearity is fixed, i.e. $Cov\left(S,f\left(S\right)\right)=1$, which simplifies model scaling. For each of the two types of non-linear mappings, we consider a version with smaller and with larger deviation from linearity, i.e. low and high values for $V\left(f\left(S\right)\right)$.

1. No non-linearity: $f\left(S\right)=S$.
2. Sigmoid non-linearity: $f\left(S\right)=\xi S^{1/3}$, and $f\left(S\right)=\xi S^{1/5}$. This means that the predicted standard scores are closer to their mean than the true scores; in other words: the standard scores overestimate the true scores when the standard scores are close to the mean, and they underestimate the true scores when the standard scores are extreme. Such non-linearity might be suspected when the latent variable is supposed to be bounded and saturates, while the experimental independent variable is unbounded or has very wide bounds. This might be the case in many areas of perception.
3. Inverse sigmoid non-linearity: $f\left(S\right)=\xi S^{3}$, and $f\left(S\right)=\xi S^{5}$. Such non-linearity might be suspected when there is evidence that small changes of the independent variable can have strong effects at extreme values. A classical example for inverse sigmoid non-linearity is subjective probability weighting in value-based decision-making: if a latent variable is manipulated by economic gambles with two fixed potential outcomes and different probabilities of winning, then the predicted and true values of the latent variable are likely to relate by inverse sigmoid mapping. (In this example, of course, this known inverse sigmoid mapping could be taken into account when using prospect theory to predict the standard values.)

#### 2.2.3 Random aberration due to imprecision: shape

**Fig. 2:**
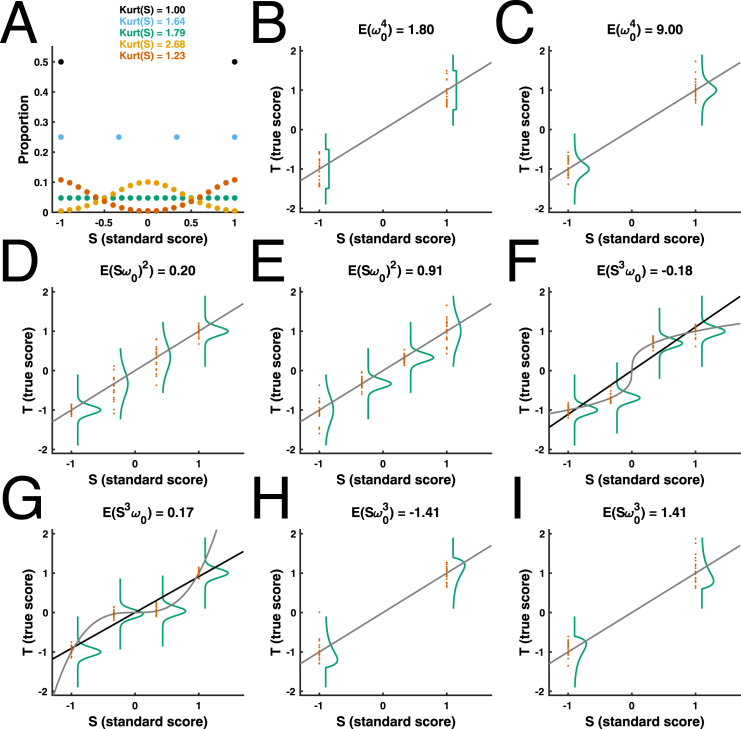
Illustration of calibration-specific terms that impact on variance of retrodictive validity estimators. A: Kurtosis of the different standard value distributions used for numeric simulations. BC: Two aberration distributions with different kurtosis. B: Continuous uniform distribution (platykurtic example). C: Student’s t-distribution with ν=5 (leptokurtic example). DE: Two random aberration distributions with different covariance of squared standard values and aberration. D: (Conditional) normal distribution with larger variance for smaller absolute values of S. E: (Conditional) normal distribution with smaller variance for larger absolute values of S. FG: Sigmoid and inverse sigmoid non-linearity. HI: Two random aberration distribution with different skewness of the aberration. H: Gamma distribution with positive skew for larger S. I: Gamma distribution with negative skew for larger S.

The parameter ξ in some of these simulations was numerically determined to ensure that $V\left(\omega_{0}\right)=1$. For all simulations, $Cov\left(S,\omega_{0}\right)=0$. These distributions were selected as they provide extreme values for some features highlighted in the analytical derivation, and they are illustrated in Fig. 2. Some tentative intuition for where these features might occur in psychology is given after each distribution; these explanations do not refer to the specific example distributions but rather to the feature values being modelled.

1. Continuous uniform distribution (platykurtic example): $\omega_{0}∼U\left(-\sqrt{3},\sqrt{3}\right)$. This might for example occur due to physical or conceptual limitations in the resolution of the independent variable, i.e. it is specified only within a range.
2. Standard normal distribution (mesokurtic example): $\omega_{0}∼N\left(0,1\right)$. This is a standard assumption in many types of research.
3. Student’s t-distribution with ν=5 degrees of freedom (leptokurtic example): $\omega_{0}∼t_{5}$. Heavy-taled distributions are seen throughout psychology.
4. (Conditional) normal distribution with larger variance for smaller absolute values of S: for fixed S, $\omega_{0}∼N\left(0,\xi \exp\right)\left(\left(-\left|S\right|\right)\right)$. This might for example occur due to floor and ceiling effects if there are person-independent boundaries on achievable true scores.
5. (Conditional) normal distribution with larger variance for larger absolute values of S: for fixed S, $\omega_{0}∼N\left(0,\xi \left|S\right|\right)$. This might happen if persons differ in their boundaries on achievable true scores, or if more extreme values are achieved by qualitatively different experimental manipulations.
6. (Conditional) gamma distribution with positive skew for larger S: for fixed S, $\omega_{0}∼S\left(\Gamma \left(2,1/\sqrt{2}\right)\right)-E\left(\Gamma \left(2,1/\sqrt{2}\right)\right)$. This could happen due to floor and ceiling effects if there are person-independent boundaries on achievable true scores.
7. (Conditional) gamma distribution with positive skew for smaller S: for fixed S, $\omega_{0}∼-S\left(\Gamma \left(2,1/\sqrt{2}\right)\right)-E\left(\Gamma \left(2,1/\sqrt{2}\right)\right)$. This might occur due to a combination of a categorical/qualitative and a quantitative mechanism in generating the true score, e.g. a percept is generated as either positive or negative in relation to a reference value, and then imbued with a specific value on the half-axes, which will have more variability into the extreme direction than towards the reference.

#### 2.2.4 Random aberration due to imprecision: scale

Simulated values for $\zeta_{I}$ were drawn from a wide range, including typical values encountered in experimental settings. These can be derived from reported retrodictive validity values in calibration experiments. Systematic research using retrodictive validity as a metric has mainly been conducted in the field of human fear conditioning. Here, retrodictive validity has typically been expressed as (Cohen’s) d for a within-subjects difference between two experimental treatments. Published values range between d=0.4 for some skin conductance analysis methods, and d=1.2 for fear-potentiated startle eye-blink (e.g. Bach & Melinscak, 2020). This corresponds to retrodictive validity estimates between (Pearson’s) r=.20 and r=.51. From Bach et al. (2020) (SI Eq. (33)), we have

(1) $$\rho_{SY}=\frac{1}{\sqrt{V\left(\omega \right)+V\left(ɛ\right)+1}},$$

and so

(2) $$V\left(\omega \right)+V\left(ɛ\right)=\frac{1}{\rho_{SY}^{2}}-1.$$

Thus, the two variances combined will take values between 24 for r=.20, and 2.8 for r=.51. Since these reported estimates derived from experiments with only two possible values of S, we can assume $f\left(S\right)=S$. If aberration and error variance were equal, this would correspond to $\zeta_{I}=3.46$ and $\zeta_{I}=1.18$, respectively. Assuming that retrodictive validity might be lower or higher in other areas of psychology, $\zeta_{I}$ was varied over the interval $\left[0.1,4\right]$.

#### 2.2.5 Measurement error

Because measurement error is not usually known a priori, and can depend on the other model quantities in complicated ways, all simulations use a placeholder error distribution that represents i.i.d. Gaussian noise with three levels: $ɛ∼N\left(0,\sigma^{2}\right)$ with $\sigma \in \left\{0.1,1,4\right\}$.

#### 2.2.6 Simulation settings

All simulations used Matlab’s default Mersenne twister to generate random numbers. Distributions were simulated by drawing $10^{7}$ samples. Asymptotic results used all of these samples to approximate the expectations in Eq. (3), from which estimator variance was analytically calculated. For finite-sample results, I randomly selected (with replacement) from these simulated distributions $10^{6}$ samples of size n=100 (based on the sample size suggested in Bach et al. (2023)) and computed the resulting variance of the sample retrodictive validity estimator. All simulation results depict the quantity $nV\left(r_{SY}\right)$, from which estimator variance is computed by division through actual sample size. For small samples, sample size can have an impact on $nV\left(r_{SY}\right)$. Hence, I compared the results to simulations with sample sizes of N=50 and N=1000. On average, the absolute differences in $nV\left(r_{SY}\right)$ between the differently sized samples was about 1%, and in more than 90% of the simulated scenarios, the absolute difference was below 5%.

## 3 Results

### 3.1 Analytical results

The asymptotic limit of the sample estimator variance can be decomposed into the following terms (see Appendix for derivation):

$$\underset{n\to \infty }{\lim}nV\left(r_{SY}\right)=$$

$$E\left(S^{4}\right)\frac{1}{4}\left(\rho_{SY}^{2}-2\rho_{SY}^{4}+\rho_{SY}^{6}\right)+$$

$$E\left(\omega^{4}\right)\left(\frac{1}{4}\rho_{SY}^{6}\right)+$$

$$E\left({\left(S\omega \right)}^{2}\right)\left(\rho_{SY}^{2}-\frac{5}{2}\rho_{SY}^{4}+\frac{3}{2}\rho_{SY}^{6}\right)+$$

$$E\left(S^{3}\omega \right)\left(\rho_{SY}^{2}-2\rho_{SY}^{4}+\rho_{SY}^{6}\right)+$$

$$E\left(S\omega^{3}\right)\left(-\rho_{SY}^{4}+\rho_{SY}^{6}\right)+$$

(3) $$+h_{ɛ}\left(ɛ\right),$$

where $h_{ɛ}$ collapses all termes involving the (a priori unknown and unknowable) measurement error. In the following I discuss the five terms not involving measurement error, keeping in mind the goal of minimising $V\left(r_{SY}\right)$. Fig. 2 illustrates the different quantities. I note (see Appendix) that although the sum of these terms is non-negative, some of them can be negative (and thus serve to reduce estimator variance).

#### Weighted kurtosis of the standard value distribution.

(4) $$A_{1}≔E\left(S^{4}\right)\frac{1}{4}\left(\rho_{SY}^{2}-2\rho_{SY}^{4}+\rho_{SY}^{6}\right)$$

(5) $$=\frac{1}{4}Kurt\left(S\right)\left(\rho_{SY}^{2}-2\rho_{SY}^{4}+\rho_{SY}^{6}\right),$$

because $E\left(S\right)=0$ and $V\left(S\right)=1$. Both factors in this term are non-negative on $\rho_{SY}\in \left[0,1\right]$. Kurtosis of the standard value distribution is under experimental control, and reducing it will always reduce $A_{1}$. Fig. 2A illustrates different distributions and their kurtosis. The second factor has minima at $\rho_{SY}=0$ and $\rho_{SY}=1$, and a maximum at $\rho_{SY}=1/\sqrt{3}\approx 0.577$ (corresponding to $V\left(\omega \right)+V\left(ɛ\right)=2$). For fixed $V\left(ɛ\right)$, this means that reducing $V\left(\omega \right)$ from a given value can increase or reduce this term. The maximum value of $A_{1}$ on the interval $\rho_{SY}\in \left[0,1\right]$ is $\frac{1}{27}Kurt\left(S\right)$. While kurtosis is theoretically unbounded, high-kurtosis distributions of S appear unnecessary in practice, and for a plausible normal distribution of S, the maximum is $A_{1}\approx 0.11$. Thus, overall this term makes a relatively modest contribution to $V\left(r_{SY}\right)$.

#### Weighted kurtosis of the aberration distribution.

(6) $$A_{2}≔E\left(\omega^{4}\right)\left(\frac{1}{4}\rho_{SY}^{6}\right)$$

(7) $$=\frac{1}{4}Kurt\left(\omega \right)\left(\frac{V^{2}\left(\omega \right)}{{\left(1+V\left(\omega \right)+V\left(ɛ\right)\right)}^{3}}\right)$$

using $E\left(\omega \right)=0$ and thus, $Kurt\left(\omega \right)=E\left(\omega^{4}\right)/V^{2}\left(\omega \right)$, and Eq. (1). Both factors in this term are non-negative on $\rho_{SY}\in \left[0,1\right]$. Reducing aberration kurtosis would thus always reduce $A_{2}$, although in practice this will usually not be under experimental control (Fig. 2BC). For fixed $V\left(ɛ\right)$, the second factor has a maximum at $V\left(\omega \right)=2V\left(ɛ\right)+2$. In words, if the aberration is relatively large compared to the measurement error, then decreasing the aberration increases $A_{2}$. If $V\left(\omega \right)$ is below this bound, then decreasing the aberration decreases $A_{2}$. This is the case for example if $V\left(\omega \right)=V\left(ɛ\right)$. Because $V\left(\omega \right)$ also impacts on the other terms, its net effect can only be understood in the numerical simulations.

#### Weighted covariance of squared intended values and squared aberration.

(8) $$A_{3}≔E\left({\left(S\omega \right)}^{2}\right)\left(\rho_{SY}^{2}-\frac{5}{2}\rho_{SY}^{4}+\frac{3}{2}\rho_{SY}^{6}\right)$$

(9) $$=E\left({\left(S\omega_{0}\right)}^{2}\right)\left(\frac{V\left(\omega \right)}{1+V\left(\omega \right)+V\left(ɛ\right)}-\frac{5V\left(\omega \right)}{2{\left(1+V\left(\omega \right)+V\left(ɛ\right)\right)}^{2}}\right)\left(\;+\;\frac{3V\left(\omega \right)}{2{\left(1+V\left(\omega \right)+V\left(ɛ\right)\right)}^{3}}\right).$$

with Eq. (1) and $\omega =\omega_{0}\sqrt{V\left(\omega \right)}$. The first factor is non-negative. If S and ω are stochastically independent, or if there are just two equiprobable values of S, then the first factor equals 1. In other cases, the first factor is large when ω takes larger absolute values for more extreme values of S (Fig. 2DE). For the second factor, if $V\left(ɛ\right)>1/2$, then reducing $V\left(\omega \right)$ will decrease $A_{2}$. If $V\left(ɛ\right)\leq 1/2$, then on an interval $\left[0,V_{m}\left(\omega \right)\right]$, decreasing $V\left(\omega \right)$ increases $A_{2}$, where $V_{m}\left(\omega \right)$ depends on $V\left(ɛ\right)$ with $V_{m}\left(\omega \right)<0.3$. The impact of changing $V\left(\omega \right)$ is analysed in detail in the numerical simulations below.

#### Weighted systematic aberration.

(10) $$A_{4}≔E\left(S^{3}\omega \right)\left(\rho_{SY}^{2}-2\rho_{SY}^{4}+\rho_{SY}^{6}\right)$$

(11) $$=E\left(S^{3}\omega_{0}\right)\left(\frac{\sqrt{V\left(\omega \right)}}{1+V\left(\omega \right)+V\left(ɛ\right)}-\frac{2\sqrt{V\left(\omega \right)}}{{\left(1+V\left(\omega \right)+V\left(ɛ\right)\right)}^{2}}\right)\left(\;+\;\frac{\sqrt{V\left(\omega \right)}}{{\left(1+V\left(\omega \right)+V\left(ɛ\right)\right)}^{3}}\right)$$

**Fig. 3:**
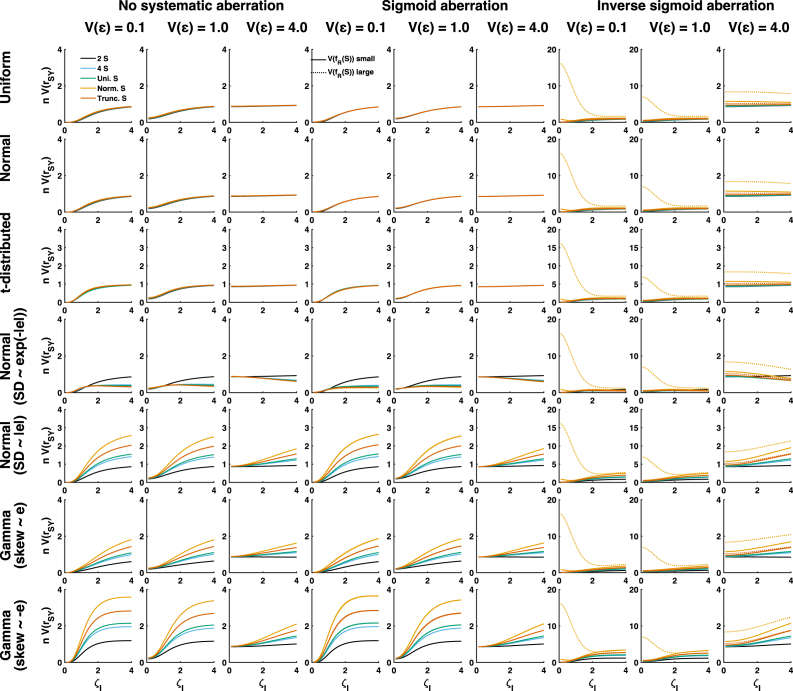
Simulated asymptotic results for 63 scenarios with different standard value distributions (colours), different random aberration distributions (rows), different scale of random aberration (x-axis), different systematic non-linearity (columns) and different levels of measurement error (columns). All panels show the product of asymptotic estimator variance with sample size. Note that under inverse sigmoid aberration, two columns are displayed with a different limit on the y-axis.

with Eq. (1) and $\omega =\omega_{0}\sqrt{V\left(\omega \right)}$. The first factor is zero if there are two or three equidistant values of E, or if there is no non-linearity, i.e., $f\left(S\right)=S$. Otherwise, the first factor is positive when ω takes more extreme values for more extreme values of S (e.g. inverse sigmoid non-linearity f, Fig. 2F), and negative in the opposite case (e.g. sigmoid non-linearity f, Fig. 2G). The second factor is non-negative. On an interval $\left[0,V_{m}\left(\omega \right)\right]$, decreasing $V\left(\omega \right)$ decreases this factor. The minimum value of $V_{m}\left(\omega \right)$ is achieved when $V\left(ɛ\right)=0$, which yields $V_{m}\left(\omega \right)=5$. It is plausible to assume that $V\left(\omega \right)$ will rarely be larger than this value. The impact of changing $V\left(\omega \right)$ then only depends on the first factor and is analysed in detail in the numerical simulations below.

#### Weighted skewness of the aberration.

(12) $$A_{5}≔E\left(S\omega^{3}\right)\left(-\rho_{SY}^{4}+\rho_{SY}^{6}\right)$$

(13) $$=E\left(S\omega_{0}^{3}\right)\left(-\frac{V^{3/2}\left(\omega \right)}{{\left(1+V\left(\omega \right)+V\left(ɛ\right)\right)}^{2}}+\frac{V^{3/2}\left(\omega \right)}{{\left(1+V\left(\omega \right)+V\left(ɛ\right)\right)}^{3}}\right),$$

with Eq. (1) and $\omega =\omega_{0}\sqrt{V\left(\omega \right)}$. The first factor is positive if ω is positively skewed for large(r) values of S and negatively skewed for small(er) values of S (see Fig. 2HI). For two values of S, this would mean that the distribution of ω has long tails into both directions, but fewer values in the middle. For the opposite situation, the first factor will be negative. If ω has no skew for any value of S, then this term will be zero. The second factor is negative. On an interval $\left[0,V_{m}\left(\omega \right)\right]$, decreasing $V\left(\omega \right)$ increases this factor. The minimum value of $V_{m}\left(\omega \right)$ is achieved when $V\left(ɛ\right)=0$ which yields $V_{0}\left(\omega \right)=5$. It is plausible to assume that $V\left(\omega \right)$ will rarely be larger than this value. The impact of changing $V\left(\omega \right)$ then only depends on the first factor and is analysed in detail in the numerical simulations below.

### 3.2 Numerical results

**Fig. 4:**
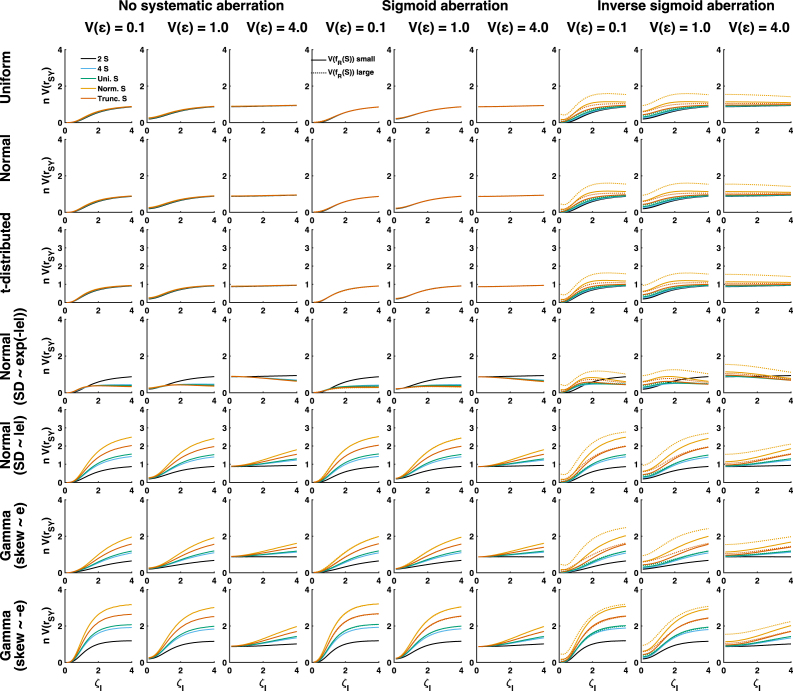
Simulated finite sample results for 63 scenarios with different standard value distributions (colours), different random aberration distributions (rows), different scale of random aberration (x-axis), different systematic non-linearity (columns) and different levels of measurement error (columns). All panels show the product of asymptotic estimator variance with sample size. This quantity was averaged across $10^{5}$ samples of size n=100.

**Fig. 5:**
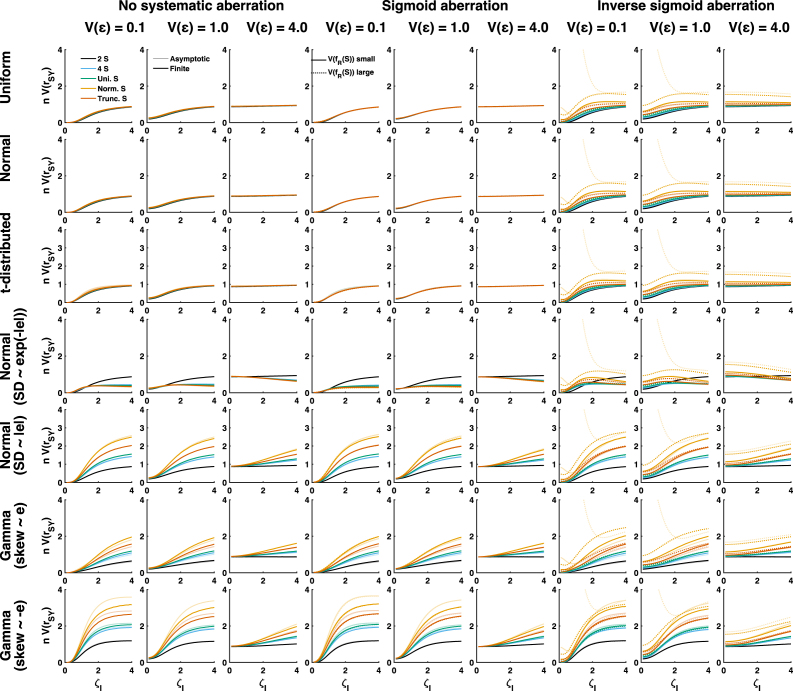
Direct comparison of asymptotic limits (Fig. 3) and finite-sample results (Fig. 4). Faint colours denote the asymptotic limits.

Because several of the analytically identified terms depend on aberration in different ways, I numerically simulated various scenarios to quantify their impact on estimator variance. Fig. 3 shows simulated asymptotic results, where rows refer to different distributions of random aberration $\omega_{0}$, columns to different systematic non-linearities and levels of measurement error $V\left(ɛ\right)$, and the x-axis to different levels of random aberration $\zeta_{I}$. Using the same layout, Fig. 4 shows simulated results from finite samples, and Fig. 5 shows the direct comparison.

We first consider estimator variance in the absence of systematic non-linearity (left three columns in Fig. 3, Fig. 4). When $\omega_{0}$ is independent of the standard values S (top three rows), different distributions of S yield relatively similar estimator variance. This confirms the analytically derived result that although the kurtosis of S does affect $A_{1}$, its numerical impact is relatively small. Furthermore, reducing the scale of aberration can reduce estimator variance considerably, but only for low to medium levels of measurement error, i.e. when the error variance $V\left(ɛ\right)$ is on a lower or same order of magnitude as the variance of the aberration. In high-noise scenarios, when measurement error variance is on a larger order, reducing aberration only has a small effect.

Next, we consider conditional distributions of $\omega_{0}$ that depend on S. Here, different choices of S yield more dissimilar estimator variance. In most situations, binary S yields the smallest, and normally distributed S the largest estimator variance, with a small impact of truncating S. In most circumstances, reducing the scale of $\omega_{I}$ reduces estimator variance. However, in the specific case that $V\left(\omega_{0}\right)$ inversely scales with the absolute values of S (fourth row), these results are reversed: binary S yields the largest estimator variance, and reducing the scale of $\omega_{I}$ can increase estimator variance, in particular if measurement error is large. For small and medium levels of measurement noise, this effect is mainly due to term $A_{2}$, as anticipated in the analytical results. However, $A_{2}$ has hardly an impact at high measurement noise. Here, the increase in estimator variance is due to an interplay of several factors, including the error variance-dependent term h. At high levels of measurement noise, this term generally increases with decreasing $\zeta_{I}$. Note that in the absence of prior knowledge on the distribution of measurement error, I assumed i.i.d. normally distributed error for the simulations. Insofar as some of these results depend on h, they may not generalise.

In the presence of sigmoid non-linearity in the aberration, the results look very similar (middle three columns), and there is almost no impact of the variance of the systematic aberration. However, inverse sigmoid aberration changes this picture (right three columns) in several important ways. First, asymptotic estimator variance becomes very large for large systematic non-linearity when S is a normal distribution and the scale of $\omega_{I}$ is small. This is due to term $A_{2}$. The implementation of inverse sigmoid aberration used here is a cubic function, and the kurtosis of the cube of a standard normally distributed variable corresponds to the value of its 12th moment which is 11!!=2079. This excessive kurtosis is reduced by adding random noise to ω; hence the phenomenon is particularly pronounced for small scale of $\omega_{I}$ and ɛ. However, kurtosis is not as excessive for any truncated S or in finite samples (see Fig. 4), and this phenomenon therefore may be irrelevant for practical purposes. The following three differences apply both for asymptotic and finite-sample cases. Secondly, estimator variance is generally much larger for (truncated) normal distribution of S than for the discrete and continuous uniform distributions, due to an increase of the term $A_{3}$. Third, the scale of the non-linearity has a larger impact on estimator variance here than for sigmoid non-linearity, again due to an impact on term $A_{3}$. Fourth, there are more scenarios in which decreasing the scale of $\omega_{I}$ increases estimator variance.

## 4 Discussion

In this note, I investigated quantities that determine the sample variance of retrodictive validity estimators. Of particular interest are design features that an experimenter can control; and it is useful to consider how different settings of these features fare under various combinations of non-controllable features.

The first feature under experimental control is the sample size, which has a well-known linear relation with estimator variance. Increasing sample size, can, in any scenario, reduce estimator variance. All other results concern the quantity $nV\left(r_{SY}\right)$, where n is the sample size. In other words, they describe how estimator variance is affected when sample size is constant. The second feature under experimental control is the distribution of S, which the experimenter can freely choose. In the absence of systematic non-linearity in the aberration, and when imprecision aberration is i.i.d., the choice of S has no large impact on estimator variance. However, in many other situations, estimator variance increases from binary to discrete to continuous uniform to normally distributed S. The third feature of interest is the scale of the aberration distribution $\zeta_{I}$, which is at least under indirect experimental control. Reducing imprecision aberration reduces estimator variance in many but not all scenarios, and it can sometimes even increase estimator variance. Because it is generally unknown which scenario occurs in a given calibration experiment, the effect of reducing imprecision aberration is difficult to predict.

From these results, several recommendations for the design of calibration experiments can be derived. First, it may be preferable to use a relatively small number of discrete experimental treatments, as is usually the case anyway in many fields of experimental psychology. Binary treatments fare particularly well, but in advanced experimental fields, a goal may be to calibrate a measurement across a range of values and to include a possible impact of systematic aberration, which would motivate the use of several discrete values. In case a continuous distribution is desired, then normal distributions, however desirable as they are for simplified statistical computations, should be avoided in favour of a continuous uniform distribution. A second recommendation relates to aberration and the use of resources. Reducing aberration is obviously desirable in its own right, but it may be costly if it involves specific experimental efforts such as shielded testing rooms, prolonged resting periods, monitoring participants over extended periods of time, etc. The present results suggest that reducing imprecision aberration is useful in most scenarios but not when measurement noise is relatively high, and not under some particular aberration distributions. Since these distributions are not a priori known to the calibration planner, if there are limited resources available they may be better invested into increasing sample size, which uniformly and linearly decreases estimator variance.

Other distributional features had a pronounced impact on estimator variance but many of these will be hard to control or even to know, for example the shape of the aberration distribution. However, as one of these features, systematic non-linearity deserves particular attention as it relates to shortcomings in the substantive theory used to generate the predicted standard values S. While not under experimental control in a particular calibration experiment, substantive theory can be improved over time, and its results can be incorporated into the analysis of a calibration experiment even after the data are acquired. A particularly problematic case, inverse sigmoid aberration, is exemplified by subjective probability weighting in value-based decision making. If a latent variable is manipulated by changing outcome probabilities in an economic gamble with two fixed potential outcomes, then the latent variable is likely to relate to the probabilities in an inverse-sigmoid manner. If, however, predicted values were derived with a linear probability weighting, then these would be misspecified with an inverse-sigmoid aberration. This could then be improved by using cumulative prospect theory to derive more accurate predictions of the true scores. Thus, reducing systematic aberration merits investment and yields long-term returns, as the results can be used retrospectively and prospectively in many calibration experiments. This requires improving substantive theory and thus resonates with recent theoretical proposals that measurement should principally be based on substantive theory (Borgstede & Eggert, 2023).

These recommendations are based on the objective of minimising estimator variance. I note that there might be other objectives when designing a calibration experiment. For example, experimental aberration constitutes an important source of measurement uncertainty (BIPM, IEC, IFCC, ILAC, IUPAC, IUPAP, ISO, OIML, 2012, Rigdon and Sarstedt, 2022, Rigdon et al., 2020). Aberration cannot be reduced by better measurement instruments. In the terminology of this article, experimental aberration places an upper limit on achievable values of retrodictive validity. This might be undesirable in itself, and as such, reducing the scale of measurement aberration might be an independent goal of calibration design, beyond reducing estimator variance.

All results are necessarily limited to the choice of scenarios analysed. To motivate these scenarios, I analytically derived distribution features that have a large impact on estimator variance, and then simulated distributions with extreme values of these features. Doing so may have missed out on some other distributions that are possibly more relevant in particular fields of experimental psychology. All simulations are openly available and can easily be expanded to encompass other situations. Furthermore, the present analysis is focused on independently randomised (rather than balanced) experimental treatments. This is a plausible experimental approach for between-subjects studies, but the case of balanced experimental treatments is particularly important for within-subjects designs. Future work will explore this experimental design in a purely numerical approach.

As a general limitation of the calibration approach, and similar to the concept of construct validity (Cronbach & Meehl, 1955), it does not allow to empirically separate trueness (bias) and precision of the measurement (Bach et al., 2020). Trueness of the measurement refers to systematic deviation from the true scores. This can occur for example due to unknown non-linearities in the mapping from true scores to measured scores (see for illustration e.g., Dunn & Anderson, 2022), or because one is measuring not the latent variable of interest but a different, but somewhat correlated variable. Precision on the other hand, relates to variability in the measurement under constant true scores. Although these can be distinguished in theory – just as one can distinguish systematic aberration (bias) from imprecision aberration – retrodictive validity collapses both into one accuracy metric. In order to distinguish the terms, there are two approaches (Bach et al., 2020). The weaker one is to analyse pairs of values of S, in which case, the influence of trueness will likely be reduced compared to a larger set of values of S. The second is to additionally evaluate reliability (Brandmaier et al., 2018), from which precision can be computed under justifiable assumptions (McDonald, 2013). Note, however, that the design of parallel tests in the context of experimental measurement can be non-trivial, as discussed previously (see supplemental information in Bach et al. (2020)). Future work will address this experimental limitation and hopefully contribute to the derivation of uncertainty metrics in experimental measurement (Rigdon and Sarstedt, 2022, Rigdon et al., 2020).

To summarise, estimator variance in calibration experiments can be reduced by using uniformly distributed, preferably discrete, experimental treatments; by reducing inverse sigmoid experimental aberration; and in many but not all circumstances by reducing imprecision aberration. If resources are limited, then the most predictable route to reducing estimator variance, under all experimental circumstances, remains increasing sample size.

## Data Availability

All simulations and code are publicly available on OSF: https://osf.io/dfg9e/.

## Appendix

The following theorem is proven in Steiger and Hakstian (1982). Let ρ denote a population (bivariate) correlation coefficient, and r the corresponding sample correlation coefficient .

Theorem 1
*For a sample with size* N *, let* n≔N−1 *. For two sample correlation coefficients with arbitrary parent distributions, the joint distribution of* $\sqrt{n}\left(r_{ij}-\rho_{ij}\right)$ *and* $\sqrt{n}\left(r_{kh}-\rho_{kh}\right)$ *converges to a bivariate normal distribution with covariance matrix* $\left[\begin{array}{cc} \gamma_{ij,ij} & \gamma_{ij,kh}\\ \gamma_{ij,kh} & \gamma_{kh,kh} \end{array}\right]$ *and*

$$\gamma_{ij,kh}=\rho_{ijkh}+\frac{1}{4}\rho_{ij}\rho_{kh}\left(\rho_{iikk}+\rho_{jjkk}+\rho_{iihh}+\rho_{jjhh}\right)-\frac{1}{2}\rho_{ij}\left(\rho_{iikh}+\rho_{jjkh}\right)-\frac{1}{2}\rho_{kh}\left(\rho_{ijkk}+\rho_{ijhh}\right)$$

*with*

$$\sigma_{ij}≔Cov\left(X_{i},X_{j}\right)$$

$$\sigma_{ijkh}≔E\left(\left(X_{i}-\mu_{i}\right)\left(X_{j}-\mu_{j}\right)\left(X_{k}-\mu_{k}\right)\left(X_{h}-\mu_{h}\right)\right)$$

$$\rho_{ij}≔\frac{\sigma_{ij}}{\sqrt{\sigma_{ii}\sigma_{jj}}}$$

$$\rho_{ijkh}≔\frac{\sigma_{ijkh}}{\sqrt{\sigma_{ii}\sigma_{jj}\sigma_{kk}\sigma_{hh}}}.$$

For the variance, this theorem reduces to:

(A.1) $$\gamma_{ij,ij}=\rho_{ijij}+\frac{1}{4}\rho_{ij}^{2}\left(\rho_{iiii}+2\rho_{iijj}+\rho_{jjjj}\right)-\rho_{ij}\left(\rho_{iiij}+\rho_{ijjj}\right)$$

(A.2) $$=\frac{\sigma_{iijj}}{\sigma_{ii}\sigma_{jj}}+\frac{1}{4}\rho_{ij}^{2}\left(\frac{\sigma_{iiii}}{\sigma_{ii}^{2}}+2\frac{\sigma_{iijj}}{\sigma_{ii}\sigma_{jj}}+\frac{\sigma_{jjjj}}{\sigma_{jj}^{2}}\right)-\rho_{ij}\left(\frac{\sigma_{iiij}}{\sqrt{\sigma_{ii}^{3}\sigma_{jj}}}+\frac{\sigma_{ijjj}}{\sqrt{\sigma_{ii}\sigma_{jj}^{3}}}\right).$$

For sample retrodictive validity $r_{SY}≔Cor\left(s,y\right)$, we have:

$$\underset{n\to \infty }{\lim}nV\left(r_{SY}\right)=\gamma_{ij,ij}=\frac{E\left({\left(SY\right)}^{2}\right)}{V\left(S\right)V\left(Y\right)}\;+\;\frac{1}{4}\rho_{EY}^{2}\left(\frac{E\left(S^{4}\right)}{V^{2}\left(S\right)}+2\frac{E\left({\left(SY\right)}^{2}\right)}{V\left(E\right)V\left(Y\right)}+\frac{E\left(Y^{4}\right)}{V^{2}\left(Y\right)}\right)$$

(A.3) $$-\rho_{SY}\left(\frac{E\left(S^{3}Y\right)}{\sqrt{V^{3}\left(S\right)V\left(Y\right)}}+\frac{E\left(SY^{3}\right)}{\sqrt{V\left(S\right)V^{3}\left(Y\right)}}\right)$$

$$=\frac{E\left({\left(SY\right)}^{2}\right)}{V\left(Y\right)}+\frac{1}{4}\rho_{SY}^{2}\left(E\left(S^{4}\right)+2\frac{E\left({\left(SY\right)}^{2}\right)}{V\left(Y\right)}+\frac{E\left(Y^{4}\right)}{V^{2}\left(Y\right)}\right)$$

(A.4) $$-\rho_{SY}\left(\frac{E\left(S^{3}Y\right)}{\sqrt{V\left(Y\right)}}+\frac{E\left(SY^{3}\right)}{\sqrt{V^{3}\left(Y\right)}}\right)$$

where the second equality used $E\left(S\right)=E\left(Y\right)=0$ and $V\left(S\right)=1$.

Now we expand the expectations involving Y=S+ω+ɛ:

(A.5) $$E\left({\left(SY\right)}^{2}\right)=E\left({\left(S\left(S+\omega +ɛ\right)\right)}^{2}\right)$$

(A.6) $$=E\left({\left(S^{2}+S\omega +Sɛ\right)}^{2}\right)$$

(A.7) $$=E\left(S^{4}+{\left(S\omega \right)}^{2}+{\left(Sɛ\right)}^{2}+2S^{3}\omega +2S^{3}ɛ+2S^{2}\omega ɛ\right)$$

(A.8) $$E\left(S^{3}Y\right)=E\left(S^{3}\left(S+\omega +ɛ\right)\right)$$

(A.9) $$=E\left(S^{4}+S^{3}\omega +S^{3}ɛ\right)$$

(A.10) $$E\left(SY^{3}\right)=E\left(S{\left(S+\omega +ɛ\right)}^{3}\right)$$

(A.11) $$=E\left(S^{4}+S\omega^{3}+S{ɛ}^{3}+3S^{3}\omega +3S^{3}ɛ+3S^{2}\omega^{2}\right)\left(\;+\;3S\omega^{2}ɛ+3S^{2}{ɛ}^{2}+3S\omega {ɛ}^{2}+6S^{2}\omega ɛ\right)$$

(A.12) $$E\left(Y^{4}\right)=E\left({\left(S+\omega +ɛ\right)}^{4}\right)$$

(A.13) $$=E\left(S^{4}+\omega^{4}+{ɛ}^{4}+4S^{3}\omega +4S^{3}ɛ+4S\omega^{3}+4S{ɛ}^{3}+4\omega^{3}ɛ+4\omega {ɛ}^{3}+\right)$$

$$\left(6S^{2}\omega^{2}+6S^{2}{ɛ}^{2}+6\omega^{2}{ɛ}^{2}+12S^{2}\omega ɛ+12S\omega^{2}ɛ+12S\omega {ɛ}^{2}\right)$$

Taking all of this together yields

$$\underset{n\to \infty }{\lim}nV\left(r_{SY}\right)=$$

$$=\frac{E\left(S^{4}+{\left(S\omega \right)}^{2}+{\left(Sɛ\right)}^{2}+2S^{3}\omega +2S^{3}ɛ+2S^{2}\omega ɛ\right)}{V\left(Y\right)}+$$

$$\frac{1}{4}\rho_{SY}^{2}\left(E\left(S^{4}\right)+2\frac{E\left(S^{4}+{\left(S\omega \right)}^{2}+{\left(Sɛ\right)}^{2}+2S^{3}\omega +2S^{3}ɛ+2S^{2}\omega ɛ\right)}{V\left(Y\right)}\right)+$$

$$\frac{E\left(S^{4}+\omega^{4}+{ɛ}^{4}+4S^{3}\omega +4S^{3}ɛ+4S\omega^{3}+4S{ɛ}^{3}+4\omega^{3}ɛ+4\omega {ɛ}^{3}\right)}{V^{2}\left(Y\right)}+$$

$$\left(\frac{E\left(6S^{2}\omega^{2}+6S^{2}{ɛ}^{2}+6\omega^{2}{ɛ}^{2}+12S^{2}\omega ɛ+12S\omega^{2}ɛ+12S\omega {ɛ}^{2}\right)}{V^{2}\left(Y\right)}\right)-$$

$$\rho_{SY}\left(\frac{E\left(S^{4}+S^{3}\omega +S^{3}ɛ\right)}{\sqrt{V\left(Y\right)}}+\right)$$

$$\frac{E\left(S^{4}+S\omega^{3}+S{ɛ}^{3}+3S^{3}\omega +3S^{3}ɛ+3S^{2}\omega^{2}\right)}{\sqrt{V^{3}\left(Y\right)}}+$$

(A.14) $$\left(\frac{E\left(3S\omega^{2}ɛ+3S^{2}{ɛ}^{2}+3S\omega {ɛ}^{2}+6S^{2}\omega ɛ\right)}{\sqrt{V^{3}\left(Y\right)}}\right).$$

Simplifying yields

$$\underset{n\to \infty }{\lim}nV\left(r_{SY}\right)=$$

$$=\rho_{SY}^{2}E\left(S^{4}+{\left(S\omega \right)}^{2}+{\left(Sɛ\right)}^{2}+2S^{3}\omega +2S^{3}ɛ+2S^{2}\omega ɛ\right)+$$

$$\frac{1}{4}\rho_{SY}^{2}\left(E\left(S^{4}\right)+2\rho_{SY}^{2}E\left(S^{4}+{\left(S\omega \right)}^{2}+{\left(Sɛ\right)}^{2}+2S^{3}\omega +2S^{3}ɛ+2S^{2}\omega ɛ\right)\right)+$$

$$\rho_{SY}^{4}E\left(S^{4}+\omega^{4}+{ɛ}^{4}+4S^{3}\omega +4S^{3}ɛ+4S\omega^{3}+4S{ɛ}^{3}+4\omega^{3}ɛ+4\omega {ɛ}^{3}\right)+$$

$$\left(\rho_{SY}^{4}E\left(6S^{2}\omega^{2}+6S^{2}{ɛ}^{2}+6\omega^{2}{ɛ}^{2}+12S^{2}\omega ɛ+12S\omega^{2}ɛ+12S\omega {ɛ}^{2}\right)\right)-$$

$$\rho_{SY}\left(\rho_{SY}E\left(S^{4}+S^{3}\omega +S^{3}ɛ\right)+\right)$$

$$\rho_{SY}^{3}E\left(S^{4}+S\omega^{3}+S{ɛ}^{3}+3S^{3}\omega +3S^{3}ɛ+3S^{2}\omega^{2}\right)+$$

(A.15) $$\left(\rho_{SY}^{3}E\left(3S\omega^{2}ɛ+3S^{2}{ɛ}^{2}+3S\omega {ɛ}^{2}+6S^{2}\omega ɛ\right)\right).$$

Now we collapse all terms involving ɛ, as they are outside the experimenter’s control, depend on the particular measurement model and thus cannot be determined a priori. This yields

$$\underset{n\to \infty }{\lim}nV\left(r_{SY}\right)=$$

$$=\rho_{SY}^{2}E\left(S^{4}+{\left(S\omega \right)}^{2}+2S^{3}\omega \right)+$$

$$\frac{1}{4}\rho_{SY}^{2}\left(E\left(S^{4}\right)+2\rho_{SY}^{2}E\left(S^{4}+{\left(S\omega \right)}^{2}+2S^{3}\omega \right)\right)+$$

$$\rho_{SY}^{4}E\left(S^{4}+\omega^{4}+4S^{3}\omega +4S\omega^{3}\right)+$$

$$\left(\rho_{SY}^{4}E\left(6S^{2}\omega^{2}\right)\right)-$$

$$\rho_{SY}\left(\rho_{SY}E\left(S^{4}+S^{3}\omega \right)+\right)$$

$$\left(\rho_{SY}^{3}E\left(S^{4}+S\omega^{3}+3S^{3}\omega +3S^{2}\omega^{2}\right)\right)$$

(A.16) $$+h_{ɛ}\left(ɛ\right).$$

Re-arranging gives:

$$\underset{n\to \infty }{\lim}nV\left(r_{SY}\right)=$$

$$E\left(S^{4}\right)\frac{1}{4}\left(\rho_{SY}^{2}-2\rho_{SY}^{4}+\rho_{SY}^{6}\right)+$$

$$E\left(\omega^{4}\right)\left(\frac{1}{4}\rho_{SY}^{6}\right)+$$

$$E\left({\left(S\omega \right)}^{2}\right)\left(\rho_{SY}^{2}-\frac{5}{2}\rho_{SY}^{4}+\frac{3}{2}\rho_{SY}^{6}\right)+$$

$$E\left(S^{3}\omega \right)\left(\rho_{SY}^{2}-2\rho_{SY}^{4}+\rho_{SY}^{6}\right)+$$

$$E\left(S\omega^{3}\right)\left(-\rho_{SY}^{4}+\rho_{SY}^{6}\right)+$$

(A.17) $$+h_{ɛ}\left(ɛ\right).$$

We note that if ɛ=0 then $h_{ɛ}\left(ɛ\right)=0$. It follows that the sum of the other terms is non-negative.
